# Hospital Practice—Case of Hip Disease, with Sudden Death from Cardiac Embolism

**Published:** 1880-04

**Authors:** 


### Selections.

### HOSPITAL PRACTICE.

*Hereford General Infirmary.—Case of Hip Disease, with sudden Death from Cardiac Embolism.* (*Under the Care of Mr. Turner.*)

For the notes of this interesting case we are indebted to Mr. Charles J. Devis, house-surgeon.

Charles H——, aged fourteen, a farmer's boy, was admitted on Nov. 21st, 1878. His illness began eight months before, when he complained of an aching pain in the right hip. He continued to work, but always with lameness. He grew worse during the two months prior to admission, and was obliged finally to leave off work. On admission he presented the symptoms and signs of synovitis of the right hip-joint—viz., flattening of the buttock, loss of the gluteal fold, flexion of the joint, and great pain on movement of the limb. There were no rigors, and no great variation of the daily temperature. The limb was extended, and Liston's long splint was applied. Iron and cod-liver oil were given internally.

On January 13th there was much tenderness and pain in the region of the hip-joint, aggravated at night, with starting of the limb when going to sleep. There was no fullness in the front of the thigh where the tenderness was greatest.

On Feb. 21st the apparatus was removed. There was much fullness over hip-joint in front of thigh, the fold of the groin being lost. There was no pain on pressing trochanters, and very little on raising the limb. The limb was very stiff at both knee and hip, and there was great œdema of whole limb. The limb was raised on a pillow and enveloped in a flannel bandage. On March

1st he was better, there being very little pain on either pressure or movement. Ordered to sit up a short time each day. On the 3rd he was obliged to return to bed on account of increase of pain and œdema of limb, which latter much resembled that of phlegmasia dolens. Extension was re-applied. On the 5th fluctuation was detected below the great trochanter over an area about two inches by one inch in extent.

On April 12th the pain was less; fluctuation not increased; œdema as great as before. Feet of bed raised on blocks, and the limb well raised on pillows.

On May 15th the œdema of limb was much less, but still persisting; the tissues were brawny and hard; there was no tenderness in the region of the femoral vein or lymphatics; the knee, to which passive motion had been applied, was a little more flexible. An aspirator needle was introduced into the abscess, the contents of which were found to be inspissated pus too thick to pass through the needle.

On June 6th complete anæsthesia was produced by ether given in Clover's inhaler, six drachms being the total quantity used. The knee was fully flexed and the hip partially so, the adhesions readily breaking down. No unusual symptom occurred during the day. Vomiting began half an hour after the operation, and continued until evening (about five hours). The patient slept well and ate his breakfast heartily at 7 o'clock on the following morning. At 8:35 a.m. he told the nurse that he had a pain in his left side, to which she replied that she would give him a fomentation until she could fetch the house-surgeon. Whilst preparing the fomentation the nurse looked up and saw the lad looking very ill, but without any breathlessness, and at once summoned the house-surgeon, who arrived at the bedside within five minutes of the first complaint of pain, only to find him at the last gasp.

*Necropsy, forty-eight hours after death.*—Hypostatic congestion and rigor mortis well marked; body well nourished; right hip almost completely anchylosed, only admitting of slight flexion; much solid œdema of right lower limb. Thorax: trunk distended with blood; pericardium fully occupied by heart; right auricle much distended, ventricle to a less degree. Lungs pale, with slight hypostatic congestion. On cutting through the pulmonary artery, it was seen to contain a colorless fibrinous clot of some consistence. Heart: Right auricle full of blood-clot, which extended through the right ventricle into the pulmonary artery to its secondary divisions; centre of clot yellow, firm, and strongly adherent to the auricular appendix; the majority of the clot softer and more friable, partly colorless and partly colored. Left heart empty; ventricle firmly contracted. Abdominal organs healthy, with the exception of a great amount of congestion, most marked in the kidneys. The disease of the hip was found confined to the articulation, the surfaces of the femur and acetabulum both being bare and rough; a large abscess directly communicating with the joint was found deeply situated among the adductor muscles. On examination of the femoral vein, this vessel was found converted into a tough fibrous cord, quite impermeable for an inch and a half in extent, the part of the vein involved being in the apex of Scarpa's triangle. On laying open the vein a firm tough clot closely united to the inner coat of the vessel was found extending upwards for half an inch, and surrounding this was some soft recent clot, which readily broke off. The remainder of the vessel, as well as the external and common iliac, was healthy and permeable.

*Remarks.*—The question that naturally arises in this case is, how much of the fatal termination was due to the disturbance of the blood-clot in the femoral vein by the manipulation given to the limb? And, depending on that, what was the sequence of events that took place in the vascular system? It would appear that on the flexion of the knee and hip-joints a portion of clot was broken off and carried into the circulation. The lodgment of this embolus in the auricular appendix gave rise to little or no disturbance, but on this was built an immense thrombus, almost filling the auricle, and extending through the ventricle into the pulmonary artery for some distance. Under these circumstances, if such they be, how is such a sudden death to be explained? Not by an extra exertion on the part of the patient, for he was at the time lying quiet in bed, and had not felt any inconvenience when having his breakfast, although occasionally reaching over to his locker.

